# Potential Role of Probiotic Strain *Lactiplantibacillus plantarum* in Control of Histamine Metabolism

**DOI:** 10.3390/biology14060734

**Published:** 2025-06-19

**Authors:** Gina Cavaliere, Egidia Costanzi, Beniamino Cenci-Goga, Marco Misuraca, Giovanna Traina

**Affiliations:** 1 Department of Pharmaceutical Sciences, University of Perugia, 06126 Perugia, Italy; gina.cavaliere@unipg.it; 2 Department of Veterinary Medicine, University of Perugia, 06126 Perugia, Italy; egidia.costanzi@unipg.it (E.C.); beniamino.cencigoga@unipg.it (B.C.-G.); marco.misuraca.tdp@gmail.com (M.M.); 3 Faculty of Veterinary Science, Department of Paraclinical Sciences, University of Pretoria, Onderstepoort 0110, South Africa

**Keywords:** *Lactiplantibacillus plantarum* LP115, diamine oxidase (DAO), HT29 cells, histamine intolerance, probiotics

## Abstract

Histamine intolerance is a condition that occurs when there is an imbalance between the accumulation and degradation of histamine within the body. Excess histamine is metabolized and then degraded by two enzymes, of which the most abundant is the vesicular diamine oxidase (DAO). An imbalance or a state of dysbiosis of the intestinal microbiota has been observed in patients with histamine intolerance compared to healthy individuals. Studies indicate that the administration of bifidobacteria or lactobacilli alone or in mixtures can alter colonic microbiota populations and metabolic activities. The present study has evaluated the ability of a probiotic bacterial strain to stimulate the release of cellular DAO from an in vitro model of the human intestinal epithelial barrier. The results indicate that, under the experimental conditions used, probiotic strain *Lactiplantibacillus plantarum* LP115 has a significant stimulatory effect on DAO secretion in adenocarcinoma cell line HT-29.

## Simple Summary

The study investigates the probiotic *Lactiplantibacillus plantarum* LP115 and its effect on histamine metabolism. In vitro tests on HT-29 intestinal cells showed that after 4 h of contact, the probiotic significantly increased the secretion of diamine oxidase (DAO), the key enzyme in degrading histamine, and reduced histamine levels in the culture medium. No toxicity was observed. The probiotic did not alter DAO protein expression, suggesting the release of preformed DAO vesicles. Findings support the potential of *L. plantarum* LP115 in managing histamine intolerance.

## 1. Introduction

Food intolerance is an abnormal non-immunological response of an organism to the intake of food or its components in quantities that are normally tolerated [1].

Histamine intolerance is a condition that occurs when there is an imbalance between the accumulation and degradation of histamine within the body. It is estimated that approximately 1% of the entire population exhibits histamine intolerance [2]. Histamine is a mediator widely present in the body. It is involved in multiple biological processes including immunomodulation, inflammatory responses during anaphylaxis, and digestive mechanisms, and it is involved in various brain functions as a neurotransmitter [3].

Histamine is a biogenic amine also present in a wide variety of foods in extremely variable concentrations. Initially, the adverse effects of the excessive intake of histamine were termed scombroid fish poisoning, but this condition is now known as histamine intoxication [1].

Subsequently, another disorder was observed to be linked to histamine intake and dependent on an enzyme deficiency [4]. The main route of the formation of histamine in food is the decarboxylation of histidine by the enzyme histidine decarboxylase, which removes the carboxyl group from histidine [5]. Fermented foods and also those contaminated by bacteria due to poor hygiene during food processing or the inadequate quality of raw materials are often contaminated by histamine generated by microorganisms with histidine decarboxylase activity [6,7]. Such decarboxylation represents a survival strategy for microorganisms in acidic environments, as well as an alternative source of energy in situations of non-optimal substrate availability [8,9].

Excess histamine is metabolized and then degraded by two cellular enzymes, the most abundant of which is vesicular diamine oxidase (DAO), followed by the cytosolic histamine-N-methyltransferase (HNMT), with the latter being more effective for circulating histamine [4].

Thus, the main barrier against exogenous histamine is represented at the intestinal level by the enzyme DAO, which prevents its passage into systemic circulation. DAO is synthesized by enterocytes and is constantly released from the intestinal mucosa into the intestine and into the blood circulation during digestion [10,11].

In humans, high levels of mRNA and DAO enzyme activity are found in the gastrointestinal tract and in the kidneys and placenta [12,13]. Plasma DAO concentrations increase by up to 150-fold during pregnancy, and this suggests that it may constitute a protective mechanism for the mother and the fetus, resulting from an excess of endogenous or exogenous histamine [14]. The inactivation of DAO using aminoguanidine in animals following exogenous histamine administration strongly supports this protective mechanism [12,15].

The clinical manifestations of histamine intolerance consist of a wide range of gastrointestinal and extraintestinal symptoms due to the ubiquitous distribution of the four histamine receptors (H1–H4) in different organs and tissues [16]. The most frequent manifestations of histamine intolerance are gastrointestinal disorders, including abdominal pain, diarrhea, and constipation.

A DAO deficiency that predisposes a certain population to intolerance may have a genetic predisposition or pathological or pharmacological origin. Previous studies have shown that subjects who show histamine intolerance have low levels of plasma DAO [17]. On the other hand, the insufficient expression of DAO (due to genetic polymorphisms) or the inhibition of its activity (due to, for example, drugs) or its unavailability increases the risk of histamine intolerance [10]. The availability of endogenous DAO activity can be compromised by the inhibition of DAO by alcohol, drugs, or other biogenic amines or by malnutrition leading to a deficiency of the enzyme cofactors vitamin B-6, copper, and vitamin C [3]. Finally, DAO deficiency can have many adverse effects on various systems, such as respiratory, cardiovascular, nervous, digestive, muscular, and skeletal [18].

Studies indicate that the administration of bifidobacteria or lactobacilli alone, in mixtures, or in combination with fermentable carbohydrates (prebiotics) can alter colonic microbiota populations and metabolic activities [19]

However, certain species of probiotic bacteria, including the *Lactobacillus reuteri* strain as well as *Lactobacillus casei* (TISTR 389) and *Lactobacillus delbrueckii* subsp. *bulgaricus* (TISTR 895), can generate histamine via the conversion of histidine using bacterial histidine decarboxylase [20].

Different reports evaluated a higher proportion of histamine-secreting bacteria (e.g., *Staphylococcus*, *Proteus*, *Clostridium perfringens*, and *Enterococcus faecalis*) in patients with histamine intolerance in comparison with a healthy control group [21]. On the other hand, among the various families of lactobacilli, *Lactiplantibacillus plantarum* (*L. plantarum*) LP115 has been found to be of particular interest as it down-regulates the immune and inflammatory mediators [22]. *L. plantarum* has been shown in human clinical trials to promote multiple beneficial effects on the immune system, combat intestinal disorders, and reduce the risk of cardiovascular disease. It is also widely used as a starter culture in food production [23,24,25].

The present study evaluates the potential of the probiotic bacterial strain *L. plantarum* LP115 to stimulate the release of endogenous DAO in an in vitro model of the human intestinal epithelial barrier.

## 2. Materials and Methods

### 2.1. Chemicals, Reagents, and Media

Dulbecco’s Modified Eagle Medium (DMEM), fetal bovine serum (FBS), trypsin-EDTA, and antibiotics (penicillin and streptomycin) were purchased from Euroclone SpA (Milan, Italy). Dulbecco’s phosphate-buffered saline, pH 7.4 (PBS), was obtained from Microgem laboratory research (Naples, Italy). Acrylamide/bis-acrylamide solution 30% (37.5:1) was obtained from Bio-Rad (Milan, Italy). Pierce^TM^ Coomassie Plus Assay Reagent was obtained from Thermo Fisher Scientific (Rockford, IL, USA). 3-[4,5-dimethyl-2-thiazolyl]-2,5-diphenyl-2-tetrazoliumbromide (MTT) was produced by Sigma-Aldrich (St. Louis, MO, USA). Chemiluminescence kit (ECL) was obtained from Millipore (Burlington, MA, USA). 

Human DAO and human histamine detection kits were purchased from Elabscience (Houston, TX, USA). Primary monoclonal antibodies anti-ABP1 [EPR24299-52] (ab278497) and anti-tubulin [YL172] (ab 6160) were obtained from ABCAM (Cambridge, UK), while HRP-linked anti-rat and anti-rabbit IgG were obtained from Thermo Fisher Scientific (Rockford, IL, USA) and from Cell Signaling Technology (Waltham, MA, USA), respectively.

### 2.2. Bacterial Strain Characterization via Acidification Curve in Skim Milk and in Whole Milk

*L. plantarum* strain 115 was used in this work [22,23]. A freeze-dried strain was grown aerobically in Nutrient Broth (NB, Oxoid CM0001, Basingstoke, UK) at 37 °C for 24 h.

To characterize the strain and asses its potential as a starter culture in cheesemaking, it was then sub-cultured in skim milk (BD Difco, Franklin Lakes, NJ, USA, 232100) at 37 °C for 24 h. The total viable cell (TVC) count (on Nutrient Agar, NA, Oxoid CM0003, incubated at 37 °C in microaerobic conditions for 24 h) was approximately log 9 cfu mL^−1^ at 24 h. For the test, the strain was inoculated into skim and whole milk after dilution to obtain an initial concentration of approximately log 7 cfu mL^−1^, which mimics the initial starter concentration in yogurt production and probiotic formulations. For the test, *L. plantarum* was inoculated as a pure culture into skim milk and whole milk at 37 °C and the pH was measured with a Foodtrode electrode (Hamilton Company, Reno, NV, USA) hooked to an Eutech pH 2700 (Eutech Instrument Europe B.V., Nijkerk, The Netherlands) which recorded pH values continuously with CyberComm 6000 (Eutech Instrument, Singapore) every minute.

To mathematically find the maximum instantaneous rate of acidification and the moment in which this is achieved, a fifth-degree polynomial equation was used as an empirical model for fitting the experimental data collected:

y = a + bx +cx^2^ + dx^3^ + ex^4^ +fx^5^ (1)

In this equation, y is the pH value, x is time (minutes), and a, b, c, d, e, and f are the regression coefficients of the independent variable x. The coefficients were determined by the statistical package STATGRAPHICS Centurion XVI version 16.2.04 (Statpoint Technologies, Inc., Warrenton, VA, USA). The first derivative of the equation gives the instantaneous acidification rate, and its maximum value (V_m_) corresponds to the inflection point of the acidification curve, whereas the second derivative gives the acceleration and one of its roots gives the x value (tm) of the inflection point. Substituting to x the tm value, the pH value corresponding to the inflection point can be evaluated using the fifth-degree equation.

### 2.3. Cell Viability

The MTT assay was used to test cell viability, as reported previously by Taticchi et al. [26]. HT-29 cells (2 × 10^4^ cells/well) were seeded with DMEM medium in 96-well plates until sub-confluence was reached. After 24 h, the culture medium was removed and replaced with fresh complete DMEM medium without antibiotics. The same culture medium was used for control experimental samples. After treatments, the medium was removed, and the cell monolayers were washed with PBS 1× to eliminate probiotics in suspension. Then, 20 µL of MTT reagent stock solution (5 mg/mL MTT in PBS 1×) was added to 180 µL of DMEM to each well. Plates were incubated for 4 h at 37 °C. Finally, the supernatant was carefully removed, and formazan salt crystals were dissolved in 100 µL DMSO at 37 °C. The absorbance of each well was recorded at 595 nm using a microplate reader (Eliza MAT 2000, DRG Instruments, GmbH, Marburg, Germany). Each experiment was performed in triplicate. Data were expressed as percentages (%) of reduced MTT relative to the control cells, assuming the absorbance of the control cells was 100%.

### 2.4. Cell Cultures and Treatment

In vitro testing was performed on a human colorectal adenocarcinoma HT-29 cell line [27,28]. HT-29 cells were maintained at 37 °C in a humidified incubator with 5% CO_2_ at 37 °C and were cultured using DMEM supplemented with 10% heat-inactivated FBS (56 °C/30 min), 100 IU/mL penicillin, and 100 µg/mL streptomycin.

Cells were maintained as a monolayer in 75 cm^2^ flasks, and the medium was replenished every 2–3 days. The cells are detached with 0.05% trypsin/0.02% EDTA for a contact time of 5 min at 37 °C and sub-cultured at a 1:3 ratio when 80% of confluence was achieved. Twenty-four hours prior to the experiment, the cell medium for intestinal cell lines was replaced with a fresh medium without antibiotics, and the latter was used throughout all of the experimental procedure. The HT-29 monolayers were treated via the addition of different concentrations of *L. plantarum* for 2 and 4 h (0.1:1 (6 × 10^3^ CFU:6 × 10^4^ HT-29); 1:1 (6 × 10^4^ CFU:6 × 10^4^ HT-29); 10:1 (6 × 10^5^ CFU:6 × 10^4^ HT-29)). After incubation, the supernatants were recovered and stored at −20 °C until DAO and histamine determination. The viability of the cells was determined as described below.

### 2.5. DAO and Histamine Determinations

To analyze the DAO and histamine concentrations, supernatants of the cells were collected and stored at −80 °C until analysis. The concentration of DAO was determined in the supernatant of cells by an ELISA test according to the manufacturer’s guidelines.

The concentration of histamine in the supernatants of the various experimental groups was determined by ELISA according to the manufacturer’s guidelines by adding 111.15 ng/mL of histamine 30 min before collecting the supernatant.

### 2.6. Western Blot Analysis

Control and treated cells were sonicated at 4 °C in lysis buffer (20 mM Tris–HCl, pH 7.5, 10 mM NaF, 150 mM NaCl, 1% Nonidet P-40, 1 mM phenylmethylsulphonyl fluoride, 1 mM Na_3_VO_4_, leupeptin (1 μg/mL), and trypsin inhibitor 10 µg/mL). Protein concentrations were estimated by the Bio-Rad protein assay using free bovine serum albumin (BSA) as the standard. An equal amount of protein, 20 μg of cell proteins, was used for SDS-PAGE electrophoresis and was electro transferred onto a nitrocellulose membrane (HIMedia Laboratories, Mumbai, India) using a Bio-Rad Transblot (Bio-Rad, Milan, Italy) [29].

Membranes were blocked for 60 min with milk buffer (1× PBS, 10% *w*/*v* non-fat dry milk, 0.1% *v*/*v* Tween-20) at room temperature and incubated overnight at 4 °C with anti-DAO (dilution 1:1000) or anti alpha tubulin (dilution 1:1000) antibodies. Western blot using anti-α-tubulin antibodies was performed to normalize the protein expression levels.

The membranes were treated with HRP-conjugated secondary antibodies for 90 min. Signals were visualized by chemiluminescence. A densitometric analysis was performed by Chemidoc Imagequant LAS500–Ge Healthcare-Life Science (Milano, Italy).

### 2.7. Statistical Analysis

Three experiments in duplicate were performed for each analysis. The data are expressed as mean ± SD. Statistical differences were investigated by one-way ANOVA coupled with a Bonferroni post hoc test. The differences were considered statistically significant at *p* < 0.05. Data analysis was performed using StatView 5.0.1 for Mac OS 9 (SAS Institute, Cary, NC, USA).

## 3. Results

### 3.1. Acidification Kinetic

**Figure 1:**
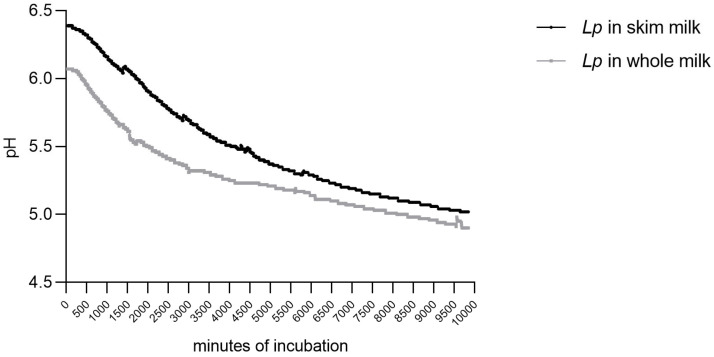
Acidification kinetics of *L. plantarum* (*Lp*) in skim and whole milk.

The parameters describing the acidification kinetics are reported in Table 1 together with the regression coefficients of the fourth-degree polynomial [Equation (1)], which was used as an empirical model. This adequately fitted the experimental data, since the r2 values varied from 0.998 to 0.999 and the actual values were almost exactly superimposed on the empirical model curves (Figure 1). The values of the maximum instantaneous acidification rate [V_m_] were of the same order for skim milk and whole milk; whole milk had the lowest V_m_ (0.0001 pH/min) and the highest tm [1555.00 min] values. The kinetic parameters for skim milk and whole milk are shown in Table 1.

### 3.2. Cell Viability

The effect of three different concentrations of *L. plantarum* on HT-29 cell viability was assessed using the MTT assay. One-way ANOVA revealed no statistically significant differences in absorbance among the treatment groups (F(2, 9) = 0.875, *p* = 0.450). The mean absorbance values (±SD) were *Lp*: HT-29 (0.1:1) [119.75 ± 37.54], *Lp*: HT-29 (1:1) [140.50 ± 38.68], and *Lp*: HT-29 (10:1) [110.50 ± 18.23].

**Figure 2:**
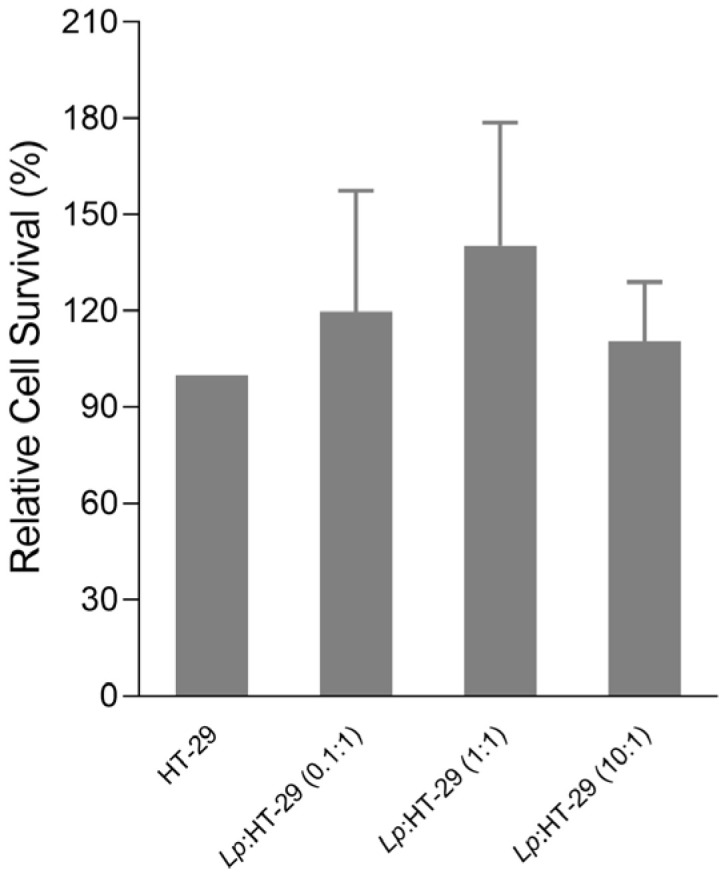
MTT results expressed as the relative cell survival. HT-29 cells were challenged using *L. plantarum* probiotics in three different colony-forming unit (CFU)/cell ratios (i.e., 0.1:1, 1:1, and 10:1) for 24 h. Statistical analysis: one-way ANOVA followed by Bonferroni post hoc, (only statistically significant differences are labeled in the chart). *Lp*: *L. plantarum*.

The results showed that the pre-treatment with *L. plantarum* at different colony-forming units (CFUs)/cells was not toxic to the HT-29 cells after 24 h, as shown in Figure 2.

### 3.3. L. plantarum Treatment Modulates DAO Release from HT-29 Cells

**Figure 3:**
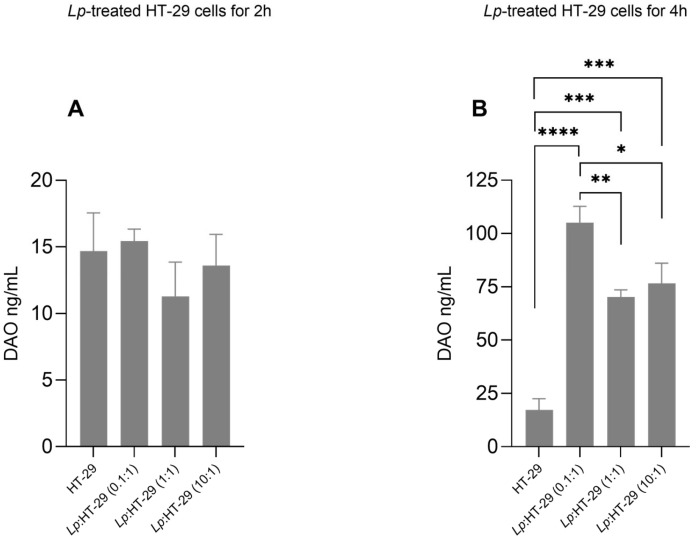
DAO levels in supernatants of *L. plantarum*-treated HT-29 cells. HT-29 cells were treated using *L. plantarum* probiotic in three different colony-forming unit (CFU)/cell ratios (i.e., 0.1:1, 1:1, and 10:1) for 2 h (**A**) and 4 h (**B**) of treatment. Data are expressed as the mean ± SD of three independent experiments. Statistical analysis: one-way ANOVA followed by Bonferroni post hoc. * *p*-value < 0.05, ** *p*-value < 0.01, *** *p*-value < 0.001, **** *p*-value ≤ 0.0001 (n = 3). (only statistically significant differences are labeled in the charts), *Lp*, *L. plantarum*.

The effects of the *L. plantarum* treatment on DAO release from HT-29 cells are shown in Figure 3. DAO concentrations in the supernatants of HT-29 cells showed no significant changes in the *L. plantarum*-treated HT-29 cells using three different colony-forming unit (CFU)/cell ratios (i.e., 0.1:1, 1:1, and 10:1) after 2 h of treatment (Figure 3A).

Conversely, DAO concentrations were significantly increased in the supernatants of *L. plantarum*:HT-29 (0.1:1), *L. plantarum*:HT-29 (1:1), and *L. plantarum*:HT-29 (10:1) cells compared to untreated cells after 4 h of treatment (Figure 3B), showing higher levels in the *L. plantarum*:HT-29 (0.1:1) cell group.

### 3.4. Histamine Concentration in HT-29 Cell Supernatants Is Affected by L. plantarum Treatment

**Figure 4:**
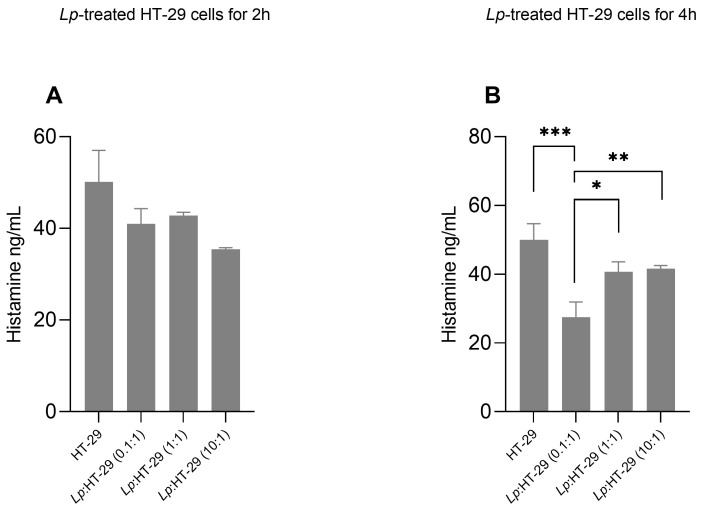
Histamine levels in supernatants of *L. plantarum*-treated HT-29 cells. HT-29 cells were treated using *L. plantarum* probiotics in three different colony-forming unit (CFU)/cell ratios (i.e., 0.1:1, 1:1, and 10:1) for 2 h (**A**) and 4 h (**B**) of treatment in the presence of histamine (115 ng/mL). Data are expressed as the mean ± SD of three independent experiments. Statistical analysis: one-way ANOVA followed by Bonferroni post hoc. * *p*-value < 0.05, ** *p*-value < 0.01, *** *p*-value < 0.001 (n = 3) (only statistically significant differences are labeled in the charts). *Lp*: *L. plantarum*.

The effects of *L. plantarum* treatment on histamine levels in supernatants of HT-29 cells are shown in Figure 4. Histamine concentrations in the supernatants of HT-29 cells showed no significant changes in the *L. plantarum*-treated HT-29 cells using three different colony-forming unit (CFU)/cell ratios (i.e., 0.1:1, 1:1, and 10:1) after 2 h of treatment (Figure 4A).

Conversely, histamine concentrations were decreased in the supernatants of *L. plantarum*:HT-29 (0.1:1), *L. plantarum*:HT-29 (1:1), and *L. plantarum*:HT-29 (10:1) cells compared to untreated cells after 4 h of treatment (Figure 4B), showing significantly lower levels in the *L. plantarum*:HT-29 (0.1:1) compared to other cell groups (Figure 4B).

### 3.5. L. plantarum Treatment on DAO Expression in HT-29 Cells

**Figure 5:**
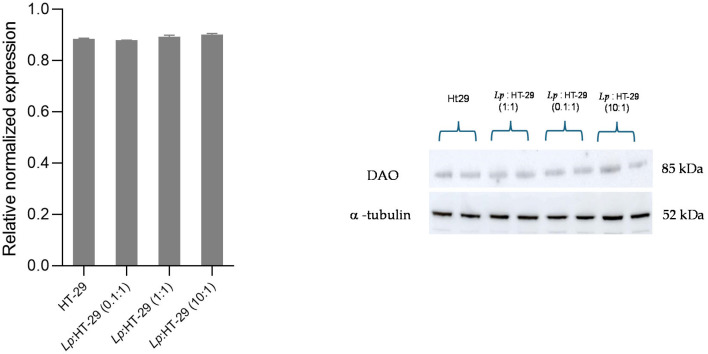
Western blot analysis for DAO protein in *L. plantarum*-treated HT-29 cells challenged using three different colony-forming unit (CFU)/cell ratios (i.e., 0.1:1, 1:1, and 10:1) after 2 h of treatment. The Western blot signals were normalized using α-tubulin as the loading control. Results are expressed as mean ± SD of the relative normalized expression. Statistical analysis: one-way ANOVA followed by Bonferroni post hoc.

**Figure 6:**
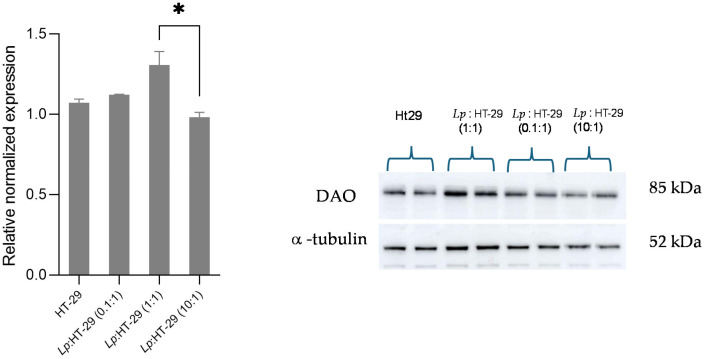
Western blot analysis for DAO protein in *L. plantarum*-treated HT-29 cells challenged using three different colony-forming unit (CFU)/cell ratios (i.e., 0.1:1, 1:1, and 10:1) after 4 h of treatment. The Western blot signals were normalized using α-tubulin as the loading control. Results are expressed as mean ± SD of the relative normalized expression. Statistical analysis: one-way ANOVA followed by Bonferroni post hoc. * *p*-value < 0.05. *Lp*: *L. plantarum*.

The changes in DAO expression in the HT-29 cells induced by *L. plantarum* were evaluated by the Western blotting analysis. The results are summarized in Figure 5 and Figure 6.

The DAO relative expression in HT-29 cells showed no significant changes in the L. plantarum-treated HT-29 cells using three different colony-forming unit (CFU)/cell ratios (i.e., 0.1:1, 1:1, and 10:1) after 2 h of treatment (Figure 5). For original images of Western blot, please refer to Supplementary Material File S1.

Regarding the analysis of DAO protein expression, although some variations are noted with respect to the different colony-forming unit (CFU)/cell ratios, these differences are not statistically significant compared to the control sample after 4 h of treatment (Figure 6).

## 4. Discussion

The present study is an important and exploratory part of a larger project focused on the ability of specific bacterial strains to stimulate the secretion of the enzyme DAO from intestinal cells as a promising and effective therapeutic approach to alleviate histamine-related dysfunctions, such as enteric ones. The main barrier against exogenous histamine is at the intestinal epithelial layer where the DAO enzyme represents a leading actor to prevent the passage of histamine into the systemic circulation. DAO inhibits the permeation of exogenous histamine through the intestinal epithelial barrier [30]. Numerous studies have shown that histamine-intolerant subjects have low levels of plasma DAO [21]. Dietary adjustments and DAO supplementation help manage symptoms for histamine-intolerant subjects [31].

A variety of studies have suggested that the influence of diet on human or animal health is mediated by altering the metabolic activity of the intestinal microbiota. In fact, the intestinal microbiota plays an important role in host health [32].

The intestinal microbiome has an enormous influence on the host’s immunological processes [33,34]. A state of dysbiosis in the intestinal microbiota has been observed in patients with histamine intolerance compared to healthy individuals [15]. A state of dysbiosis can promote inflammation of the intestinal mucosa [35]. Since histamine-degrading DAO is synthesized by mature enterocytes and stored in mucosal epithelial cells, it is plausible to believe that damage to the epithelial cells caused by an inflammatory state may lead to decreased DAO synthesis [10].

A physiological function of the DAO enzyme includes the regulation of the inflammation processes [36]. Thus, the level of DAO in the intestinal mucosa is probably linked to a state of intestinal dysbiosis that determines an alteration of the structure and composition as well as of the intestinal microbial diversity, which translates into functional changes [37]. Histamine-intolerant subjects present a certain abundance of bacteria of the phylum Proteobacteria, which, on the other hand, is a sign of dysbiosis and a marker of epithelial dysfunction [38]. In histamine-intolerance subjects, the abundance of bacteria classically associated with gut health, such as Prevotellaceae, Ruminococcus, Faecalibacterium, and *Faecablibacterium prausnitzii*, was lower, while that of histamine-secreting bacteria, such as *Staphylococcus* spp. and *Proteus* spp., various unidentified genera belonging to the family Enterobacteriaceae, and the species *Clostridium perfringens* and *Enterococcus faecalis*, was significantly higher. It is likely that this leads to the accumulation of high levels of histamine and its absorption into plasma, triggering adverse health effects [21].

It is well-known that Lactobacilli are one of the dominant species in the gut and likely affect the metabolic reactions occurring in the intestine [39]. In addition, healthy people showed a higher abundance of the Bifidobacteriaceae family.

DAO deficiency may also be an acquired condition. Inflammatory conditions affect mucosal integrity [40] and result in altered DAO activity. It is also important to note that some medications, such as proton pump inhibitors and nonsteroidal anti-inflammatory drugs, potentially inhibit DAO activity [41,42].

Some bacterial strains also possess an enzyme that ensures the endogenous synthesis of histamine. The presence of such bacteria in the gastrointestinal tract may increase the sensitivity to ingested histamine for some subjects [43].

Several studies have shown the great potential of specific bacterial strains in controlling a wide variety of functional activities, including antigenotoxic, anti-inflammatory, and antioxidant activities [44,45,46].

The aim of this study was to investigate whether the specific bacterial strain *L. plantarum* LP115, which has been shown to play interesting roles in immune mediation, can modulate the activity of DAO. Evidence has reported that *L. plantarum* can attenuate anxiety-related behavior and stress-induced intestinal dysbiosis and improve intestinal health [47,48]. The choice of this strain was also dictated by the fact that *L. plantarum* is a probiotic strain that is very versatile and can effectively colonize the human gastrointestinal system. It exhibits acid tolerance, and it has good adhesion capabilities to the intestinal mucosa [49]. Specifically, *L. plantarum* LP115 has been identified as non-histamine-producing [50].

The acidification kinetics observed for this strain indicate its strong acidifying activity in both skim and whole milk, with values comparable to those reported for *Lactococcus lactis* SL03 [51], a well-known cheesemaking starter culture. Given its ability to rapidly acidify milk, this strain demonstrates potential for use in cheesemaking applications, contributing to pH reduction and curd formation, which are essential in dairy fermentation processes [52,53].

In the present study, two and four hours were chosen as the contact times between the probiotic *L. plantarum* LP115 and intestinal cells, times that are in line with the kinetics of DAO activity [12].

Factors that monitor and influence probiotic activity include the attachment time, which refers to the time required for probiotic bacteria to successfully adhere to a surface, in this case intestinal epithelial cells, and establish a stable presence. This process involves many factors, including the type of probiotic strain, the nature of the surface, and the environmental conditions. In vitro studies typically use incubation times ranging from 30 min to several hours to assess the attachment [54]. In addition, probiotics communicate with the host by modulating key signaling pathways to enhance or rather suppress activation and influence downstream pathways. Probiotics produce a plethora of bioactive factors that influence the culture medium.

Previous studies have already suggested that secretory activity in in vitro models using probiotics and intestinal epithelial cells occurs after 4 h of contact [27,28]. The results show that in the presence of *L. plantarum* LP115, intestinal cells are stimulated to secrete DAO, as an increase in DAO is detected in the incubation medium with intestinal cells at 4 h of treatment. A model for DAO release from intestinal epithelial cells has been proposed in which DAO is released from vesicles at the basolateral membrane in response to the presence of nutrients or heparin administration and is rapidly cleared into the circulation [55]. It is likely that two hours is not enough time for these events to materialize [27,56]. This can explain why in the present study, DAO was detected in the milieu after 4 h and no DAO secretion was observed after two hours, a time evidently too short for effective adhesion and modulation signaling pathways.

To confirm that DAO was secreted by intestinal epithelial cells following treatment with *L. plantarum* LP115, whether histamine added to the culture medium was degraded was analyzed. The data presented show that with *L. plantarum* LP115, the amount of histamine is reduced. Using thew ELISA test, it is observed that the ratio *L. plantarum*:HT-29 0.1:1 is the one that determines a greater degradation of histamine. It is likely that the ratios *L. plantarum*/HT-29 10:1 and *L. plantarum*/HT-29 1:1 acidify the environment, creating non-optimal conditions for the detection of DAO levels [57,58]. Therefore, it is plausible to hypothesize that a less acidic environment allows for the detection of a higher concentration of DAO and of greater activity of DAO. This evidence is also supported by the ELISA test for histamine, where it is observed that the ratio *L. plantarum*:HT-29 0.1:1 is the one that determines a greater degradation of histamine.

Furthermore, DAO protein expression was assessed after 2 and 4 h of treatment of intestinal epithelial cells with *L. plantarum*. The results reported that the analysis of DAO protein expression at the tissue level did not show any significant changes between treated and control samples. It is likely that *L. plantarum* LP115 stimulated the secretion of preformed and available vesicles containing DAO, having detected an increase in the enzyme in the culture medium, as confirmed by the degradation of histamine in the milieu, but did not affect the protein expression at the cellular level. It is likely that the contact times tested are not sufficient or that the action of the probiotic is limited to controlling the release of the available vesicles only.

The data presented here confirm that, in the presence of exogen histamine, *L. plantarum* LP115 appears to stimulate DAO secretion by the epithelial intestinal cells, leaving us to speculate probiotic in vivo effects on histamine degradation.

## 5. Conclusions

Our findings suggest that gut microbiota control may be an important and conditioning factor in histamine intolerance. Future studies will evaluate whether contact of the intestinal epithelial cells with the probiotic affects gene expression. Further investigations will also be able to verify the persistence of bacterial vitality in the juices of the gastrointestinal canal [59]. Finally, additional experimental conditions will be considered, including a longer contact time between probiotics and intestinal cells.

## Figures and Tables

**Table 1:** Acidifying activity of *L. plantarum* as pure culture (37 °C) in sterile skim milk and sterile whole milk. Kinetic parameters and regression coefficients of the acidification curves determined by Equation (1).

Kinetic Parameters	Skim Milk	Whole Milk
Vm (ΔpH/min)	0.0003 pH/min	0.0001 pH/min
tm (min)	670.00 min	1555.00 min
pHm	6.22	5.06
Regression coefficients		
a	5.106701 × 10−20	8.252549 × 10−20
b	−1.322753 × 10−15	−2.113651 × 10−15
c	1.124665 × 10−11	1.839810 × 10−11
d	−1.918666 × 10−8	−5.828699 × 10−8
e	−2.596777 × 10−4	−4.335897 × 10−6
f	6.402713	5.152338
R2	0.9991	0.9542

V_m_ is the maximum instantaneous acidification rate, while tm and pHm are the time and the pH at which V_m_ occurred.

## Data Availability

The original contributions presented in this study are included in the article/Supplementary Material. Further inquiries can be directed to the corresponding author(s).

## Supplementary Materials

The following supporting information can be downloaded at https://www.mdpi.com/article/10.3390/biology14060734/s1, File S1: Original images of Western blot.
