# Supplementary figures and images for: Potential Role of Probiotic Strain Lactiplantibacillus plantarum in Control of Histamine Metabolism
Source: Biology (Basel). 2025 Jun 19;14(6):734. doi: 10.3390/biology14060734 (PMC12189723; doi:10.3390/biology14060734)

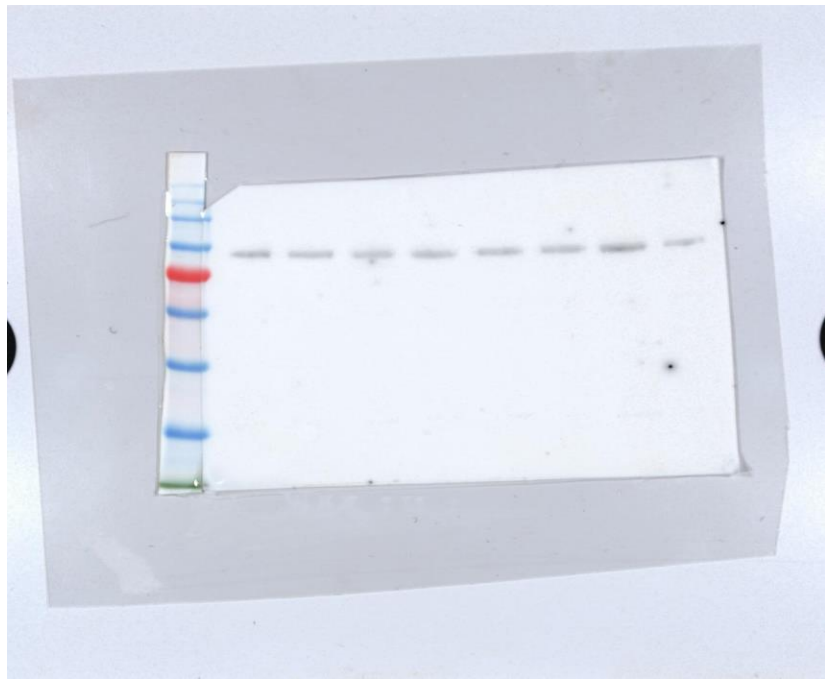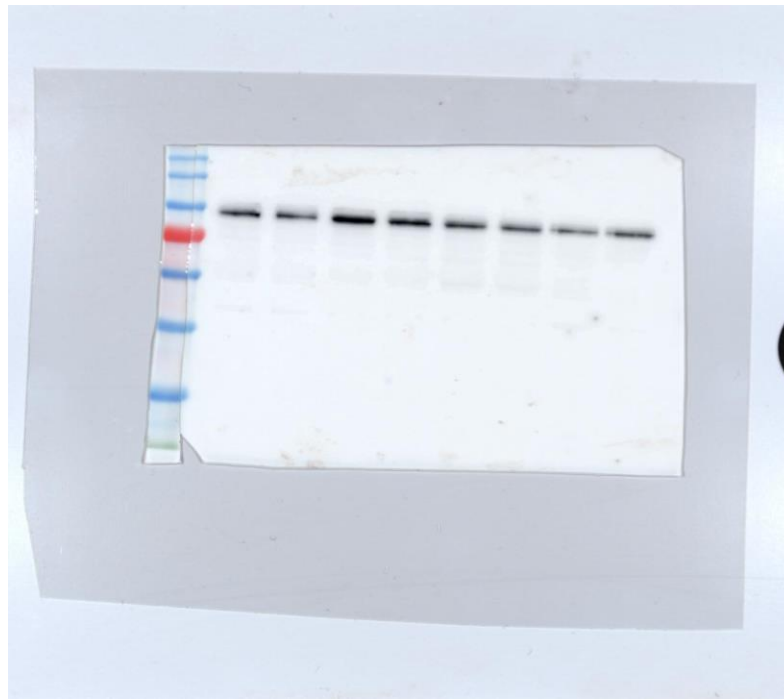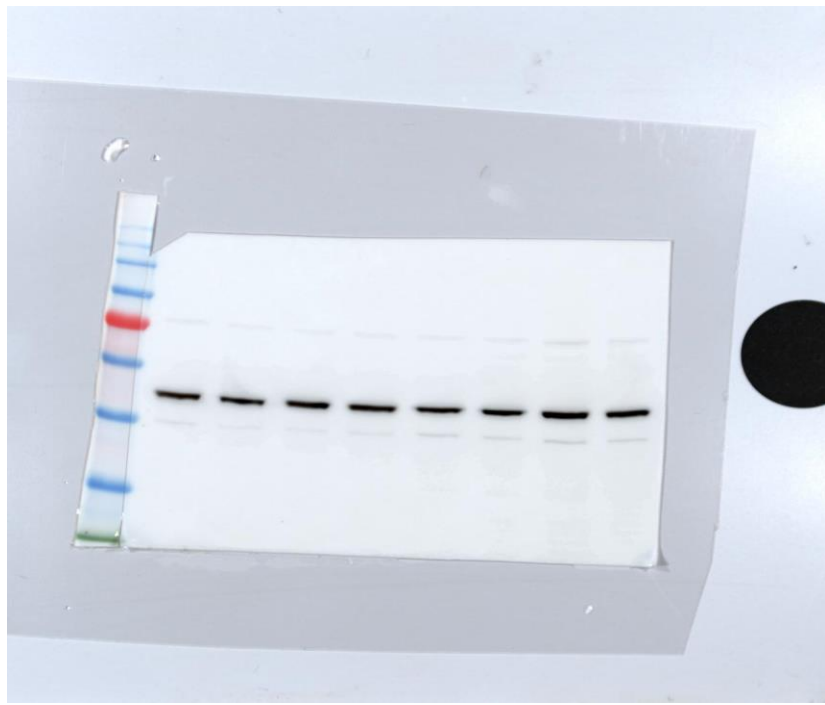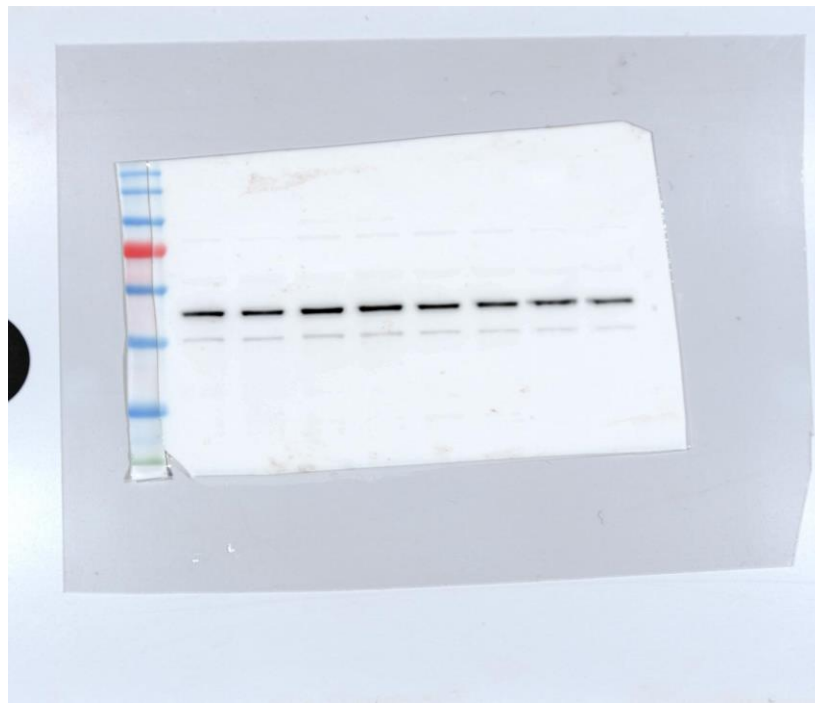

Supplement: Supplementary file 1 [file biology-14-00734-s001.zip › biology-3615140-supplementary.pdf]
